# Mr. W. Chamberlaine

**Published:** 1822-09

**Authors:** 


In August, aged 75, Mr. W. CHAMBERLAINE, surgeon and apothecary, of Aylesbury-street, Clerkenwell, formerly Secretary to the Society for the Relief of Widows and Orphans of Medical Men in London and its Vicinity, and one of the original promoters and earliest members of that society. Mr. C. was also the author of two or three little medical tracts.

